# Spatial variability of microbial assemblages associated with a dominant habitat-forming seaweed

**DOI:** 10.3389/fmicb.2015.00230

**Published:** 2015-03-26

**Authors:** Alexandra H. Campbell, Ezequiel M. Marzinelli, Jon Gelber, Peter D. Steinberg

**Affiliations:** ^1^Centre for Marine Bio-Innovation and School of Biological, Earth and Environmental Sciences, University of New South WalesSydney, NSW, Australia; ^2^Sydney Institute of Marine Science, MosmanNSW, Australia

**Keywords:** seaweed-microbe interaction, biofilm, colonization, succession

## Abstract

Macroalgal surfaces support abundant and diverse microorganisms within biofilms, which are often involved in fundamental functions relating to the health and defense of their seaweed hosts, including algal development, facilitation of spore release, and chemical antifouling. Given these intimate and important interactions, environmental changes have the potential to negatively impact macroalgae by disrupting seaweed–microbe interactions. We used the disappearance of the dominant canopy-forming fucoid *Phyllospora comosa* from the metropolitan coast of Sydney, NSW, Australia as a model system to study these interactions. We transplanted *Phyllospora* individuals from nearby, extant populations back onto reefs in Sydney to test whether bacterial assemblages associated with seaweed surfaces would be influenced by (i) the host itself, independently of where it occurs, (ii) the type of habitat where the host occurs, or (iii) site-specific differences. Analyses of bacterial DNA fingerprints (terminal fragment length polymorphisms) indicated that assemblages of bacteria on *Phyllospora* were not habitat-specific. Rather, they were primarily influenced by local, site-specific conditions with some evidence for host-specificity in some cases. This could suggest a lottery model of host-surface colonization, by which hosts are colonized by ‘suitable’ bacteria available in the local species pool, resulting in high variability in assemblage structure across sites, but where some species in the community are specific to the host and possibly influenced by differences in host traits.

## Introduction

Marine macroorganisms live in persistent contact with diverse microorganisms that are abundant and ubiquitous in the surrounding seawater (Reinheimer, 1992) and within biofilms on their surfaces (Wahl, 1989). Although our understanding of the functional importance of biofilm-associated microorganisms in the lives of the higher organisms they live upon is still evolving (Egan et al., 2008), emerging evidence points to their fundamental involvement in the development, functioning, and defense of diverse macroorganisms (e.g., Armstrong et al., 2001; Lindquist et al., 2005; Wahl et al., 2012).

The phylogenetic structure of biofilm assemblages often has a high degree of organismal- (e.g., Longford et al., 2007), species- (e.g., Taylor et al., 2004), and tissue-specificity (e.g., Thiel et al., 2007; Campbell et al., 2011; Fernandes et al., 2012). More recent work has also provided evidence for functional redundancy within microbial consortia in biofilms (Burke et al., 2011a), where various combinations of phylotypes are capable of providing a core set of functions as required by the host (Burke et al., 2011b).

Functional redundancy within microbial biofilms could be beneficial to host organisms that rely on them for development, function, or defense, if a disturbance disrupts the composition of a biofilm, because lost functions could be restored by functionally (but not necessarily taxonomically) similar microbes available in the local species pool. Marine ecosystems are undergoing rapid change on a global scale, with rates of ocean warming higher than on land (Burrows et al., 2011), and worldwide increases in coastal development (Small and Nicholls, 2003; Bullieri and Chapman, 2010) leading to widespread degradation in coastal marine ecosystems (Jackson, 2001; Lotze et al., 2006; Airoldi and Beck, 2007). Environmentally mediated impacts have already affected diverse marine macroorganisms at multiple spatial scales, including species distributional range shifts (Parmesan, 2006), habitat fragmentation (Goodsell et al., 2007), higher incidences of disease (Harvell et al., 1999), and species extinctions (Sala and Knowlton, 2006). Evidence suggests that environmental change is also having profound and alarming effects on the abundance (Sarmento et al., 2010), distribution (Cook et al., 2011), and function (Wohlers et al., 2009) of planktonic marine microorganisms. However, we know almost nothing about how environmental change can affect the composition or function of epibiotic, biofilm-associated microorganisms (Wahl et al., 2012).

Much of the available information on environmental impacts on host–biofilm interactions comes from studies into coral holobionts (Mouchka et al., 2010). The composition (Ben-Haim et al., 2003; Bourne et al., 2007) and functional gene profile (Thurber et al., 2009) of coral-associated microbial assemblages can change with changes in the environment. Higher temperatures are often correlated with the detection of pathogenic strains within coral-associated microbial communities (Ben-Haim et al., 2003; Bourne et al., 2007), the production of lytic compounds (Ben-Haim et al., 2003), or the up-regulation of genes involved in pathogenicity (Thurber et al., 2009). The involvement of bacteria (and other microorganisms) in coral bleaching and subsequent coral reef decline is now a major focus of environmental microbiology, given the ecological and socio-economic importance of these ecosystems.

On temperate coasts, macroalgae are the dominant habitat-forming primary producers, playing analogous ecological roles to corals on tropical reefs (Steneck et al., 2002). Similar to corals, large, canopy-forming macroalgae are declining from many temperate rocky reefs (e.g., Steneck et al., 2002; Airoldi and Beck, 2007; Connell et al., 2008; Wernberg et al., 2011), but these systems receive far less attention than their tropical counterparts. Consequently, our understanding of the importance of bacteria to macroalgae and the effects of environmental change on macroalgal–bacterial interactions is less well developed (see Egan et al., 2012; Minich and Dinsdale, 2014). However, recent work highlights the importance of bacteria to the development (e.g., Marshall et al., 2006), function (e.g., de Oliveira et al., 2012), and defense (Egan et al., 2008) of some seaweeds. Like corals, there is evidence that seaweed-associated bacterial communities can be species-specific (Lachnit et al., 2009), fluctuate seasonally (Lachnit et al., 2011), and as a function of host condition (Campbell et al., 2011; Fernandes et al., 2012).

When investigating microbes associated with a green alga (*Ulva australis*) Burke et al. (2011a) found a high degree of within-species variability with respect to biofilm composition, but conservation of the functional gene profiles of microbial assemblages among samples. Their findings suggest macroalgae like *U. australis* rely on functions provided by their surface biofilms. Thus, any environmentally mediated disruption to the biofilm could have negative impacts for the macroalga, if lost functions cannot be restored rapidly. Although seasonally correlated changes in macroalgal-associated biofilms have been recorded (Lachnit et al., 2011) and some laboratory experiments have demonstrated that the composition of biofilms associated with macroalgae are affected by environmental factors [e.g., temperature, Stratil et al. (2013); and salinity, Stratil et al. (2014)], these ideas have not been tested experimentally in the field.

We experimentally investigated how changing an alga’s environment affected the composition of its surface biofilms, using a large, brown fucoid alga, *Phyllospora comosa* (hereafter ‘*Phyllospora*’). Like many other large canopy-forming macroalgae, *Phyllospora* is showing signs of decline and has disappeared from *ca.* Seventy kilometers of coastline adjacent to the metropolitan area of Sydney, NSW, Australia’s largest city, but remains dominant on shallow subtidal reefs north and south of the city (Coleman et al., 2008). The local disappearance of this species was linked to poor water quality in the region due to sewage pollution. Since the 1980s, sewage treatment has improved and deep ocean outfalls have been constructed, which has vastly enhanced water quality in the region (Scanes and Philip, 1995). Despite this improvement, *Phyllospora* has failed to recover and remains absent in this area. Genetic analyses of populations of *Phyllospora* north and south of this range fragmentation suggest a high degree of connectivity and genetic exchange across its gap in distribution. We hypothesized that environmental conditions adjacent to Sydney might affect *Phyllospora*-associated biofilms and that this may be one factor that has contributed to its failure to re-establish naturally in the region. To investigate this, we transplanted *Phyllospora* individuals from extant populations back onto reefs within the Sydney region and compared the bacterial communities that developed.

## Materials and Methods

### Field Experiments

To determine whether bacterial communities on *P. comosa* individuals were (i) host-specific (i.e., that all *Phyllospora* individuals will have similar communities, regardless of where they occur or are moved to); (ii) influenced by a particular environment (i.e., will change when they are moved from a ‘*Phyllospora*’ to a ‘non-*Phyllospora’* habitat); or (iii) site-specific (i.e., they will change when they are moved to a particular place, regardless of whether it is a ‘*Phyllospora*’ habitat or not), we transplanted adults from two extant populations on the periphery of Sydney (donor habitats) into two physically similar reef habitats within metropolitan Sydney where *Phyllospora* has been absent for several decades (recipient habitats; Coleman et al., 2008; Campbell et al., 2014).

Full details of the experimental design and procedure can be found in Campbell et al. (2014) but briefly, the donor populations on the periphery of Sydney were in Cronulla (Cr; 34^∘^03^′^23^′′^ S 151^∘^09^′^23^′′^ E) and Palm Beach (PB; 33^∘^35^′^58^′′^ S 151^∘^19^′^43^′′^ E). Shallow rocky reefs at these places are characterized by a mosaic of patches of *Phyllospora* forests (size-range: 7–40 m^2^), barrens, turfing corallines, and ‘fringe’ habitats, with few individuals of the kelp *Ecklonia radiata*. The recipient habitats in Sydney were in Long Bay (LB; 33^∘^57^′^58^′′^ S 151^∘^15^′^27^′′^ E) and Cape Banks (CB; 33^∘^59^′^57^′′^ S 151^∘^14^′^52^′′^ E). Reefs at these recipient sites are very similar to those in donor places, except that patches of *Ecklonia* forests are more abundant and *Phyllospora* forests are absent. Collections and transplantations were carried out under a Scientific Collection Permit (# P00/0054-6.0) issued to the authors by the New South Wales Department of Primary Industries (Fishing and Aquaculture).

Experiments were done twice. In the first experiment (February 28 to May 9, 2011), 40 adults were collected haphazardly (collected individuals were typically 1–3 m apart) at the same depth (1–2 m) from each donor habitat by carefully detaching the holdfast from the substratum. Individuals were kept in 50 L containers with seawater for ∼2–3 hs during transportation until reattachment.

*Phyllospora* individuals from the two donor habitats were randomly allocated to one of three treatments (as per Campbell et al., 2014): (i) Transplanted individuals (‘TP’;* n* = 20), which were moved to one of the recipient habitats in Sydney (individuals from Cronulla were moved to Long Bay, while those from Palm Beach were moved to Cape Banks); (ii) Disturbed individuals (‘D’; *n* = 10), which were disturbed in the same manner as required for transplantation, but were returned to their original donor habitat; or (iii) Translocated individuals (‘TL’; *n* = 10), which were similarly disturbed, but were taken to the other donor site (i.e., an environment in which extant, natural populations of *Phyllospora* persist – from Cr to PB and *vice versa*). Undisturbed individuals (‘U’; *n* = 20) were haphazardly selected and marked *in situ* at each donor site but otherwise not handled. Disturbance and translocation treatments allowed for us to distinguish between the effects of transplantation to a different environment from the possible effects of the transplantation procedure or the effects of simply moving the algae from one place to another, regardless of environment (Marzinelli et al., 2009).

Algae that were removed from the substratum (TP, D, and TL individuals) were re-attached using cable-ties to 0.25 m^2^ plastic meshes, which were 0.5–2 m apart and had been previously attached to bare rock in barren patches approximately at 1–2 m depth. Five individuals were attached to each mesh to approximate natural densities (mean density 6.7 ± SE 1.1 per 0.25 m^2^), creating a patch of ∼4–5 m^2^ area at each place. After 2 months, one blade from each of 2–5 *P. comosa* individuals from each treatment at each location was sampled. Sampling size varied among treatments because several individuals were lost during the experiment. Blades were selected at random from the algae and cut approximately 30 cm from the tip of the blade. Each blade was sealed underwater in an individual plastic, press-seal bag. Blades were taken to the surface, rinsed with filter-sterilized seawater to remove any unattached epibionts (Millipore 0.2 μm filter) and using a sterile cotton swab, microbial assemblages from the algal surfaces were sampled (approximately 10 cm^2^ of thallus surface was gently swabbed for 30 s). The cotton tip of each swab was aseptically transferred into individual sterile 2.0 ml cryogenic storage tubes. Tubes were closed the flash frozen onsite in liquid nitrogen then stored at -80^∘^C until processing.

The experiment was repeated in late winter/spring (started August 9, 2011). In this second experiment, algae from both donor populations were transplanted to each recipient site to test for differences between algae from different sources at the same destination. Sixty algae were collected from each donor place (Palm Beach and Cronulla) and randomly assigned to three treatments: (i) individuals TP to Long Bay (*n* = 20 from each donor site), (ii) individuals TP to Cape Banks (*n* = 20 from each donor site), (iii) TL individuals to the other donor place (*n* = 20). U individuals (*n* = 20) were haphazardly selected and marked *in situ* (four sub-patches of five individuals each to resemble replication in the other treatments). Algae were attached to meshes as described above. Total patch-sizes ranged between 4 and 8 m^2^ at each place. Bacteria on blades from each of five individuals were sampled after 5 months (January 17, 2012) as described above.

### DNA Fingerprinting of *Phyllospora*-Associated Bacterial Assemblages

To compare the composition of bacterial assemblages from *Phyllospora* in different treatments, we used a polymerase chain reaction (PCR) based DNA fingerprinting technique (terminal restriction fragment length polyorphisms [TRFLP; Liu et al., 1997]). TRFLP is popularly used by molecular ecologists to characterize and compare the composition and diversity of microbial communities (Walker et al., 2004), theoretically with a species resolution, but typically to the widely accepted ‘operational taxonomic unit’ (OTU; Nocker et al., 2007). To carry-out a TRFLP analysis, phylogenetic marker genes within the sample DNA were amplified using PCR with a fluorescent dye attached to the 5^′^ end of the forward primer. PCR products were then digested using restriction enzymes, which resulted in DNA fragments of variable length. These fragments were then physically separated in sequencing capillaries and the labeled terminal fragments were detected using a laser, producing an electropherogram. A size-standard labeled with a different fluorophore was also analyzed, allowing the fragment lengths to be estimated with a resolution of one lase pair (Liu et al., 1997; Nocker et al., 2007). Each OTU is represented by a different fragment length and thus the composition and diversity of the community can be estimated based on the polymorphism of the terminal restriction fragment lengths from a sample of community DNA. Swab samples were thawed on ice. DNA from each sample was extracted and isolated using the Powersoil DNA Isolation Kit (Mo Bio Laboratories #12888-100) then stored in a -20^∘^C freezer.

### PCR and Fragment Analysis

A fragment (∼500 bp) of the bacterial 16S rRNA gene was amplified in each sample using the community DNA as a template. Primers 27F and 519R (sequences 5^′^-AGAGTTTGATCMTGGCTCAG-3^′^ and 5^′^-GWATTACCGCGGCKGCTG-3^′^, respectively), which encompass the 16S Variable regions V1–V3, were used. Primers were fluorescently labeled on the 5^′^ end with phosphoramidite dyes (27F labeled with 6-FAM and 519R labeled with VIC; Applied Biosystems).

The PCR reaction mixtures (25 μl) contained 5 pmol of the labeled 27F and 519R primers, 12.5 μl Econotaq 2x MasterMix (Lucigen), 3 μl of community template DNA, and molecular grade H_2_O. DNA amplification was performed with a PCR Express thermal cycler (Thermo Hybaid) using the following program: a 3 min start at 94^∘^C, 30 cycles consisting of 94^∘^C denaturation for 30 s, 56^∘^C annealing for 30 s, and 72^∘^C extension for 5 min. The program continued with a final extension at 72^∘^C for 5 min. Successful amplification was verified by gel-electrophoresis of 2 μl PCR products on 1% agarose gels with gel red, visualized under UV-light. PCR products were purified and concentrated using the DNA Clean and Concentration -5 Kit (Zymo Research, D4014). Purified product was eluted in 12 μl molecular grade H_2_O and quantified using a nano-drop ND1000 (Thermo Scientific).

Terminal fragment length polymorphisms was conducted using the restriction enzyme HAEIII (NE Biolabs) and standard methodology. Fragments were visualized by fluorophore color of 6-FAM (blue) and VIC (green). TRFLP data was first analyzed using Peak Scanner (Applied Biosystems). Fragment size was determined by comparison to internal size standard Liz-600, and fragments of <30 or >600 bp were excluded. Using the program T-REX (BMC Bioinformatics), background noise was removed to distinguish true fragments from background fluorescence.

### Statistical Analyses

Bacterial TRFs data were compared among *Phyllospora* from different treatments using permutational multivariate analyses of variance (Anderson, 2001) with the PERMANOVA add-on in PRIMER v6 (Anderson et al., 2007). Similarity matrices based on Bray–Curtis distances of square-root transformed relative abundances or on Jaccard distances (presence/absence) were generated for the analyses, which used 9,999 permutations of residuals under a reduced model. Multivariate dispersion, which is an estimate of variance used to test for homogeneity among groups, was also compared among treatments (for both relative abundances and presence/absence of TRFs) using the PERMDISP analysis within the PERMANOVA add-on in PRIMER v6 (Anderson et al., 2007). *P*-values were calculated using 9,999 permutations. To visualize multivariate patterns in bacterial TRFs assemblages, non-metric multi-dimensional scaling (nMDS) was used as an ordination method using PRIMER v6 (Clarke and Gorley, 2006).

To test hypotheses about influences of the host, the environment, or the sites on surface-associated bacterial communities, we analyzed the data from the point of view of the origin of the algae and also their destination. Analyses from the point of view of the origin of the algae had two factors: Treatment, which was fixed with four levels (first experiment: U, D, TL, TP; second experiment: U, TL, TP to LB, TP to CB), and Place of origin, random, with two levels (Cr, PB). In addition, comparisons of surface-associated bacteria between algae that ended up in the same place of destination were also conducted. Analyses from the point of view of the destination of the algae in the first experiment had two factors: Treatment, fixed with three levels (U, D, TL), and Place of destination, random with two levels (Cr, Pb). Two analyses from the point of view of the destination were done for the second experiment. The first analysis was similar to that of the first experiment, with two factors: Treatment, fixed with two levels (U, TL), and Place of destination, random with two levels (Cr, Pb). The second analysis compared bacteria on algae originally from different donor populations that ended up in the same Sydney metro destination: treatment was a fixed factor with two levels (TP from PB, TP from Cr), and Place of destination was random with two levels (CB, LB). All analyses are detailed in each Table.

The model that surface-associated bacterial communities are influenced by the host leads to the prediction that the structure and composition of bacterial communities on translocated algae to sites within the *Phyllospora* habitat and on those transplanted to the Sydney metro habitat will not differ from undisturbed algae at the site of origin. Under the model of differences in the environment, we predict that bacterial communities on transplanted algae to the Sydney metro habitat will differ from those translocated to sites within the *Phyllospora* habitat, which will remain similar to undisturbed algae at the site of origin. Finally, the model that communities are site-specific leads to the prediction that the communities on translocated algae to sites within the *Phyllospora* habitat and on those transplanted to the Sydney metro habitat will differ from each other and from undisturbed algae at the site of origin, and will become similar to algae in the same place of destination.

## Results

**FIGURE 1:**
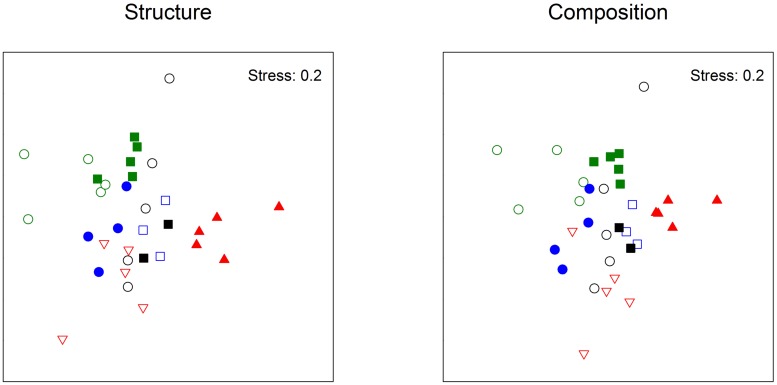
**nMDS based on Bray-Curtis measure of square-root transformed relative abundances (structure) or Jaccard measure (composition) of bacterial TRFLP on Phyllospora comosa originally from Cronulla (empty symbols) or Palm Beach (filled symbols) in the first experiment.** Treatments: Undisturbed (green symbols), Disturbed (black symbols), Translocated (blue symbols), Transplanted (red symbols). Destination places: Cronulla (circles), Palm Beach (squares), Long Bay (downward triangle), Cape Banks (upward triangle).

We detected a total of 795 microbial TRFs in this study. TRF sizes were searched against the RDP and SILVA databases using MICA (Shyu et al., 2007). No fragment was found to correspond with the predicted size of chloroplast or plastid 16S rRNA genes contained within the two databases. This indicates that the TRFLP profiles are not contaminated by host DNA or epiphytic algae and thus contain only bacterial or archaeal 16s rRNA gene information. In the first experiment, the structure and composition of bacterial fingerprints differed among treatments, but these differed according to the transplants place of origin (i.e., there was Treatment × Place interaction, **Table 1A**). Bacteria on algae transplanted from Palm Beach to Sydney metro differed from those on individuals that remained in Palm Beach undisturbed, on disturbed individuals returned to Palm Beach and on those individuals translocated to Cronulla. Although bacteria on translocated algae differed from those on undisturbed algae, there were no differences between the translocated and disturbed treatments (**Table 1A**). Algae originally from Cronulla had different bacterial TRFLP across all treatments (**Table 1A**; **Figure 1**).

**Table 1:** PERMANOVAs based on Bray–Curtis (BC) similarity measure for square-root transformed relative abundances or Jaccard similarity measure (composition) of bacterial TRF profiles associated with* Phyllospora* in the first experiment from the point of view of **(A)** the origin of the algae or **(B)** the destination.^∗^

		Square-root transformed BC			Jaccard		
Source	*df*	MS	pseudo-*F*	*p*(perm.)	MS	pseudo-*F*	*p*(perm.)

**(A) Origin**

Treatment (Tr)	3	2456	1.06	0.426	3149	0.95	0.569
Place (Pl)	1	1608	2.13	**0.002**	2462	1.68	**0.004**
Tr × Pl	3	2326	3.08	**<0.001**	3324	2.27	**<0.001**
Residual	26	756			1463		

Pairwise tests		PB: TP ≠ TL = D ≠ U Cr: TP ≠ TL ≠ D ≠ U			PB: TP ≠ TL = D ≠ U Cr: TP ≠ TL ≠ D ≠ U

**(B) Destination**

Tr	2	2041	1.35	0.291	2728	1.23	0.330
Pl	1	2272	3.13	**< 0.001**	3942	2.74	**< 0.001**
Tr × Pl	2	1517	2.09	**0.001**	2226	1.55	**0.002**
Residual	18	725			1440		

Pairwise tests		PB: TL = D ≠ U Cr: TL ≠ D ≠ U			PB: TL = D ≠ U Cr: TL = U ≠ D

*^∗^**(A)** Treatment was fixed with four levels (U, Undisturbed; D, Disturbed; TL, Translocated; TP, Transplanted), Place of origin was random with two levels (CR, Cronulla; PB, Palm Beach). **(B)** Treatment was fixed with three levels (U, D, TL), Place of destination was random with two levels (Cr, PB). The replicates were the *Phyllospora* individuals (*n* = 2–5). *P*-values were calculated using 9,999 permutations under a reduced model. Bold indicates statistical significance (at alpha = 0.005).*

When we compared bacterial fingerprints from the point of view of the site of destination of transplants, algae that ended up in PB differed from those that ended up in Cr (**Table 1B**). At each destination place, bacteria on translocated algae were, however, different from those on undisturbed algae, except at PB where translocated algae originally from Cr did not differ from disturbed algae (which originated in Palm Beach; **Table 1B**). There were no differences in dispersion of the structure or composition of bacterial TRFs among treatments (PERMDISP: *F*_3,30_ = 1.92, *p* = 0.24; *F*_3,30_ = 1.18, *p* = 0.49, respectively; **Figure 1**).

**FIGURE 2:**
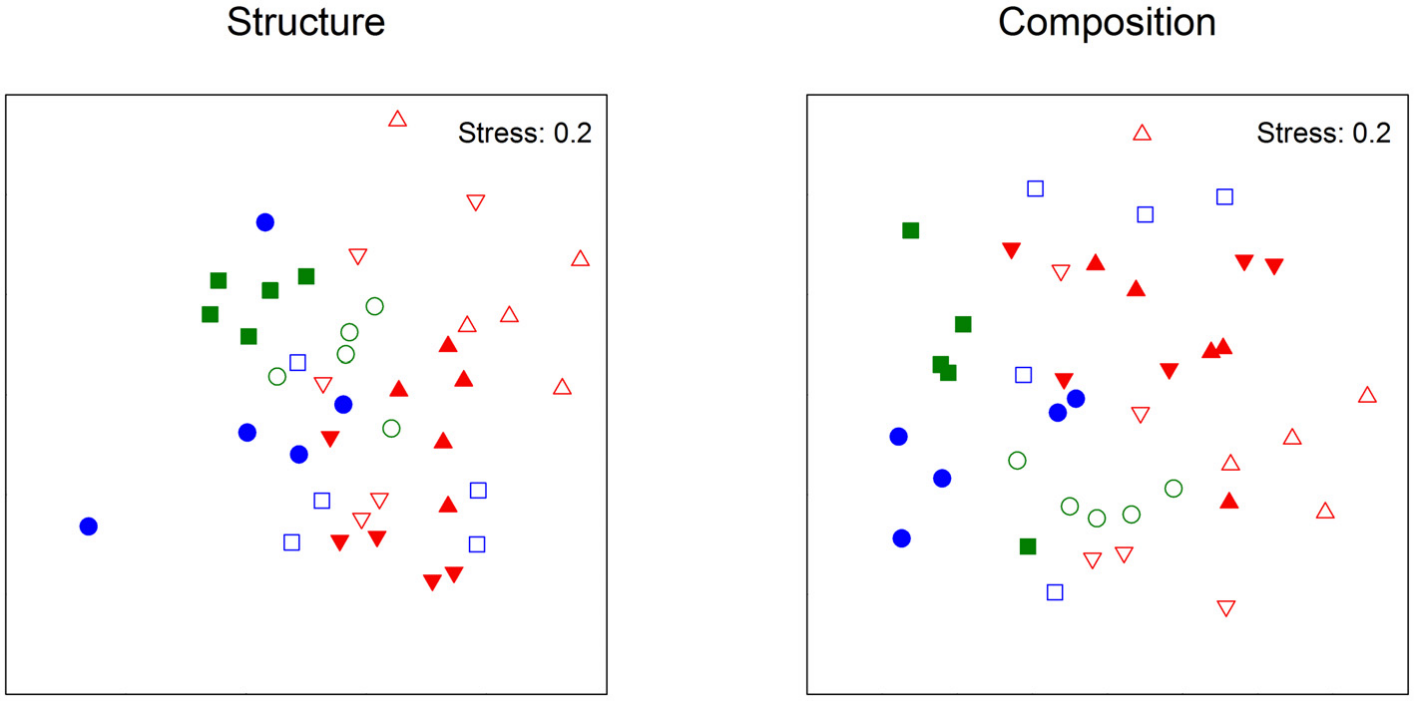
**nMDS based on Bray-Curtis measure of square-root transformed relative abundances (structure) or Jaccard measure (composition) of bacterial TRFLP on Phyllospora comosa originally from Cronulla (empty symbols) or Palm Beach (filled symbols) in the second experiment.** Treatments: Undisturbed (green symbols), Translocated (blue symbols), Transplanted (red symbols). Destination places: Cronulla (circles), Palm Beach (squares), Long Bay (downward triangle), Cape Banks (upward triangle).

In the second experiment, bacterial TRFs on algae originally from Palm Beach differed across all treatments. The same trend was observed for bacteria on algae originally from Cronulla, although pairwise comparisons could not resolve where these differences occurred and hence there was a significant Treatment × Place interaction (**Table 2**; **Figure 2**).

**Table 2:** PERMANOVAs based on BC similarity measure for square-root transformed relative abundances or Jaccard similarity measure (composition) of bacterial TRFs on *Phyllospora* in the second experiment from the point of view of the origin.^∗^

		Square-root transformed BC			Jaccard		
Source	*df*	MS	pseudo-*F*	*p*(perm.)	MS	pseudo-*F*	*p*(perm.)
Tr	3	4048	1.85	0.076	4539	1.55	0.093
Pl	1	2302	2.41	**0.001**	3512	2.01	**< 0.001**
Tr × Pl	3	2192	2.29	**<0.001**	2933	1.68	**<0.001**
Residual	32	956			1746		

Pairwise tests		PB: TP–CB ≠ TP–LB ≠ TL ≠ U Cr: TP–CB ≠ TL ≠ U TP–LB ≠ TP–CB ≠ TL U = TP–LB			PB: TP–CB ≠ TP–LB ≠ TL ≠ U Cr: TP–CB ≠ TL ≠ U TP–LB ≠ TP–CB ≠ TL U = TP–LB		

*^∗^Treatment was fixed with four levels (U, Undisturbed; TL, Translocated; TP–CB, Transplanted to Cape Banks; TP–LB, Transplanted to Long Bay), Place of origin was random with two levels (Cr, Cronulla; PB, Palm Beach). The replicates were the *Phyllospora* individuals (*n* = 5). *P*-values were calculated using 9,999 permutations under a reduced model. Bold indicates statistical significance (at alpha = 0.005).*

Bacterial TRFs on algae that ended up in PB differed from those that ended up in Cr (**Table 3A**). At each destination place, however, bacteria on translocated algae remained different from those on undisturbed algae (**Table 3A**). Bacteria on transplanted algae also differed between destination places in Sydney metro; however, bacteria on algae transplanted from Palm Beach differed from those on co-occurring algae transplanted from Cronulla at both destination places (**Table 3B**; **Figure 2**). There were no differences in dispersion of the structure or composition of bacterial TRFs among treatments (PERMDISP: *F*_3,36_ = 1.63, *p* = 0.28; *F*_3,36_ = 1.29, *p* = 0.34, respectively).

**Table 3:** PERMANOVAs based on BC similarity measure for square-root transformed relative abundances or Jaccard similarity measure (composition) of bacterial TRF profiles associated with *Phyllospora* at their destination place in **(A)** donor populations or **(B)** Sydney metro in the second experiment.^∗^

		Square-root transformed BC			Jaccard		
Source	*df*	MS	pseudo-*F*	*p*(perm.)	MS	pseudo-*F*	*p*(perm.)
**(A)**
Tr		2744	1.40	0.254	3578	1.32	0.259
Pl		2929	3.20	**< 0.001**	3732	2.19	**< 0.001**
Tr × Pl		1966	2.15	**0.004**	2702	1.58	**0.004**
Residual		914			1706		

Pairwise tests		PB: TL ≠ U Cr: TL ≠ U				PB: TL ≠ U Cr: TL ≠ U

**(B)**

Tr		2327	2.35	**0.004**	3126	1.74	**0.006**
Pl		4288	4.33	**< 0.001**	4987	2.79	**< 0.001**
Tr × Pl		879	Pooled		1875	Pooled	
Residual		997			1786		

*^∗^**(A)** Treatment was fixed with two levels (U, TL), Place of destination was random with two levels (Cr, PB). **(B)** Treatment was fixed with two levels (TP from Palm Beach or Cronulla), Place of destination was random with two levels (CB, LB). The replicates were the *Phyllospora* individuals (*n* = 5). *P*-values were calculated using 9,999 permutations under a reduced model. Non-significant interaction terms with *p* > 0.25 were pooled. Bold indicates statistical significance (at alpha = 0.005).*

## Discussion

To our knowledge, this is the first field manipulation of hosts which assesses impacts of environmental change on microbial communities associated with a large, habitat-forming macroalga. We found that in most cases, *Phyllospora*-associated microbial communities were more strongly and consistently affected by local conditions (i.e., were site-specific) than the type of environment or habitat they occurred in (i.e., ‘*Phyllospora*’ vs. ‘non-*Phyllospora*’ habitat). In the first experiment, algae that were moved from Palm Beach seemed to respond to a change of habitat (into the Sydney region) rather than to a specific site, but this pattern was not observed in individuals moved from Cronulla in the same experiment, or those from either site in the second experiment, despite some handling or ‘disturbance’ effects. This high degree of site-specificity (and lack of consistent ‘environment/habitat’ effect) suggests that simply moving algae to a different place independent of the habitat, will result in changes of their bacterial communities. We also found some evidence for host-specificity in *Phyllospora*-associated biofilms: algae translocated from one site to another did not adopt communities similar to undisturbed individuals at the destination site. Furthermore, communities on transplanted individuals with different origins that ended-up at the sample place, still supported different communities even after 5 months.

The lack of consistency in microbial communities associated with *Phyllospora* across all treatments and sites in our study does not agree with some previous studies, which compared different algal hosts and found consistent, species-specific microbial communities among places, and seasons (Lachnit et al., 2009). However, Lachnit et al. (2009, 2011) used a method with lower resolution than TRFLP fingerprinting (denaturing gradient gel electrophoresis; DGGE). Although in many studies, TRFLP and DGGE fingerprinting techniques yield similar results (Smalla et al., 2007; Campbell et al., 2011; Fernandes et al., 2012), TRFLP is a more sensitive technique (Moeseneder et al., 1999; Enwall and Hallin, 2009), and so smaller differences will be better detected in fingerprints generated by this method than DGGE, particularly in ecological studies with multiple factors.

Furthermore, Lachnit et al. (2009) compared microbial communities from several algal species. Differences among species may outweigh site- or treatment-specific differences within a single species. We did not include an ‘out-group’ (i.e., comparison with a different species or water-borne microorganisms), in part because such comparisons in our experience – particularly when using surrounding seawater as the comparator – reveal such large differences as to be uninformative with respect to the macroalgal communities (Burke et al., 2011b and below). However, comparisons with other algae could have provided a larger conceptual scale against which to compare *Phyllospora* individuals from different sites and/or treatments, and such comparisons are currently underway.

In contrast, Burke et al. (2011b) compared microbial communities from replicate samples of the green alga *U. australis* occurring in separate rock pools at a single site and also compared algal-associated communities to those within the surrounding water column, by creating and sequencing sophisticated 16S rRNA gene clone libraries. As well as finding almost no similarity between algal-associated and water-borne microbial communities, they detected a very high degree of variability among communities associated with algal samples from different rock pools (despite the ‘out-group’ comparison with water samples). They proposed that microorganisms could colonize algal surfaces using a ‘competitive lottery model,’ in which multiple species could colonize algal surfaces, so long as a core set of functions (rather than a core phylogeny) was represented within the microbial consortia. Indeed, there was a high degree of conservation of functional gene profiles expressed by the microbial communities from replicate algal samples (Burke et al., 2011a). Interestingly, previous work on the same system using the lower-resolution technique DGGE, suggested that a temporally and spatially consistent, species-specific community was present on *U. australis* (Tujula et al., 2010). In the latter study, a core set of OTUs was always present, despite some spatial and temporal variation. Our results suggest that *Phyllospora* may require a core set of functions from its biofilm, rather than a core set of ‘species’ (or OTUs) and that local, site-specific conditions rather than habitat-type will influence the phylogenetic composition of *Phyllospora*’s biofilm.

However, because translocated individuals did not come to resemble undisturbed individuals and furthermore, because transplanted individuals from different origins did not conform to a ‘destination site-specific’ community, this suggests some degree of host-specificity – that is, microbial communities are influenced by more than simply the site in which they occur (or to which they are moved). This may simply be an issue of timing: longer than 5 months may be required for convergence of microbial assemblages to occur in some cases. This seems unlikely, however, given the relatively rapid rates of colonization and succession in microbial communities compared to macroorganisms. Alternatively, the starting conditions of the biofilm may influence microbial succession such that multiple, alternative stable states (rather than just one) are possible, given a set of environmental conditions and host traits. Such concepts have been widely discussed and tested in the field of classical ecology (reviewed by McCook, 1994; Young et al., 2001) and are beginning to feature in the study of microbial ecology as well (Burke et al., 2011a). The fact that there was some degree of host-specificity suggests that a component of the community may be influenced by differences in host traits. Host genetic variation can strongly influence the structure of microbiomes associated with plants (e.g., Bulgarelli et al., 2013) and animals, including mice (Benson et al., 2010) and humans (Arumugam et al., 2011). Our results suggest that *Phyllospora* may also have a core microbiome that can be influenced by local, site-specific conditions, and host traits.

Because crayweed remains absent from the Sydney region, we hypothesized that the environment in Sydney may impact seaweed-associated microbial communities, which may, in turn, have contributed to its failure to recover in the region. We found no consistent evidence that the environment within Sydney had any significant impact on *Phyllospora*-associated microbes. Previously, we reported that individuals transplanted from extant populations to these recipient sites within the Sydney region had survivorship rates comparable to those in natural populations (Campbell et al., 2014). Together with these observations, this suggests that environmental conditions within Sydney are now suitable to support *Phyllospora* again. Furthermore, they suggest that individuals transplanted to or recruiting naturally onto reefs in Sydney will not develop a ‘Sydney-specific’ biofilm, rather they will adopt a biofilm specific to the local conditions (whether it is within or outside of the Sydney region).

It is possible, however, that at the time of its disappearance, the habitat within Sydney had a negative impact on *Phyllospora*’s interactions with its biofilm. Then, a high volume of poorly treated sewage was released adjacent to the shorelines where this species was dominant (Coleman et al., 2008). Ecotoxicological experiments with *Phyllospora* suggest that it is physiologically sensitive to high levels of nutrients (Burridge et al., 1995). The production of metabolites and other natural products by macroalgae strongly influences the composition and maintenance of biofilms on their surfaces (Egan et al., 2012). Thus an environmentally mediated change in physiology (e.g., due to high levels of pollution) might have indirectly altered *Phyllospora*’s surface-associated microbial community, which may have contributed to is decline. Understanding the role of microorganisms in the health of important, habitat-forming organisms is essential to understand the processes that affect their persistence, and to inform efforts to restore populations of these organisms if they decline. The emergence of molecular techniques to rapidly (and with increasing affordability) assess the composition of microbial assemblages associated with these organisms and even characterize their functional gene profiles, facilitates the inclusion of microbes into more ecological studies.

Most studies on holobionts to date have shown either strong host-specificity of surface-associated microbial communities, suggesting that hosts require a core set of specific taxa, or high variability of surface-associated microbial communities, suggesting that hosts may be colonized by taxonomically distinct bacteria available in the local species pool and where a core set of specific functions is more important for the host than a core set of specific taxa. Our results suggest that a combination of both processes influence bacteria on the *Phyllospora* holobiont. Thus, although some component of the community may vary across sites depending on the available taxa, other components of the community may be driven by specific traits of the host.

## Conflict of Interest Statement

The authors declare that the research was conducted in the absence of any commercial or financial relationships that could be construed as a potential conflict of interest.
